# Associations between eHealth literacy, mental health-seeking attitude, and mental wellbeing among young electronic media users in China during the COVID-19 pandemic

**DOI:** 10.3389/fpubh.2023.1139786

**Published:** 2023-02-24

**Authors:** Richard Huan Xu, Xiao-lu Bao, Lu-shao-bo Shi, Dong Wang

**Affiliations:** ^1^Department of Rehabilitation Sciences, The Hong Kong Polytechnic University, Kowloon, Hong Kong SAR, China; ^2^JC School of Public Health and Primary Care, The Chinese University of Hong Kong, Shatin, Hong Kong SAR, China; ^3^School of Health Management, Southern Medical University, Guangzhou, China; ^4^Institute of Health Management, Southern Medical University, Guangzhou, China

**Keywords:** mental help-seeking, mental wellbeing, structural equation modeling, young people, eHealth literacy

## Abstract

**Objective:**

This study aimed to examine the associations among mental health related eHealth literacy (eHL), mental health-seeking attitude, and wellbeing among Chinese young electronic media users during the COVID-19 pandemic.

**Methods:**

A web-based cross-sectional survey was conducted in Guangzhou, China. The modified eHealth literacy Scale, Mental Help-Seeking Attitudes Scale, and Short Warwick-Edinburgh Mental Wellbeing Scale were used. Structural equation modeling (SEM) examined the associations between them and was adjusted by several controlled variables.

**Results:**

Totally, 1,008 participants completed the questionnaire and provided valid responses. The eHL showed a statistically significant and direct effect on mental wellbeing in this sample. The higher the level of eHL, the better wellbeing of the participants. The mental health-seeking attitude is also positively correlated with mental wellbeing, indicating that the more positive attitude toward seeking mental health services, the better the wellbeing participants reported. The higher level of eHL is significantly associated with a more positive attitude toward seeking mental health services.

**Conclusion:**

Training to improve eHL may optimize young electronic media users' mental health outcomes. Development and use of a mental health specific eHL instrument in future studies should be encouraged.

## Introduction

With the coronavirus disease (COVID-19), the new normality of maintaining social distancing is rapidly changing how people interact with family members, friends, and communities. Virtual interactions are more frequently used than in-person communication, and web-based video chat is rapidly replacing public gatherings worldwide (1). This is also an emerging trend in healthcare, given most COVID-19 patients and their close contacts are under lockdown and healthcare providers are at infection risk. Although there are multiple solutions for providing healthcare services, ehealth is being embraced like never before, which is identified as an effective way to use existing technologies to facilitate optimal service delivery while minimizing the hazard of direct person-to-person interaction (2). Specifically, the rapid use of portable digital devices enables healthcare information and services can be provided through the Internet with a higher volume and speed. Hence, people increased their usage of the Internet and reported it as a major source of healthcare information and services during the pandemic (3).

Greater exposure to COVID-19, such as being infected or quarantined, significantly impacted public mental health (4). For example, a study in Hong Kong found that the mental health of more than one-quarter of respondents had deteriorated (5). In a Spanish study, individuals in quarantine reported a significantly higher distress level and more mental health problems than the general public (6). Another US study revealed that there is a significant and consistent correlation between COVID-19 and enhanced rates of psychiatric disorders (7). Moreover, the World Health Organization also indicated that people, specifically young adults, are highly likely to suffer from mental health problems because of the new realities of working from home, temporary unemployment, home-schooling of children, and lack of physical contact with other family members (8). It is essential to provide psychological interventions to prevent mental health problems and maintain access to mental health services for people during the pandemic. As most mental health care service users were unable to continue physically accessing mental health support because of strict social distancing rules, the Internet has been identified as a valuable alternative for providing information and services to improve mental wellbeing among young adults (9). A French study also exhibited that seeking mental health-related information and support on the Internet is a relatively common behavior among young adults during COVID-19 (10).

Although it is increasingly recognized that managing mental health is as important as physical health, previous studies reported that people, specifically, young adults are reluctant to seek professional treatment. For instance, despite nearly one-quarter being diagnosed with psychological problems to some extent, Philips et al. found that <10% of Chinese people sought mental health services (11). Another study also reported that around 70% of Chinese adolescent students who reported suffering from depression did not seek professional mental health help (12). Providing help-seeking for young adults in need should ensure the availability of mental health support, but this is a major challenge for mental health services (13). Recent studies have revealed that web-based interventions may help overcome barriers (e.g., anonymity and privacy) reported by adolescents and young adults in the traditional face-to-face psychological consultation (14, 15).

Although Internet-mediated therapy and support have benefits, young adults' knowledge and skills to seek and use reliable web-based information to manage their mental health are unknown. Mental health related eHL, a concept derived from eHealth literacy and mental health literacy, refers to the knowledge and skills to use web-based information to obtain and maintain positive mental health, understand problems and their treatments, avoid stigma related to problems, and enhance help-seeking efficacy (16, 17). Improving mental health literacy can facilitate self-identified depression, reduce perceived barriers to help-seeking, and improve mental wellbeing (18, 19). However, empirical evidence about the impact of mental health-related eHL on mental wellbeing is rarely reported. A US study indicated that parents with low eHL were more likely to have a child with a high risk for developing a mental health disorder (20). Another study found that there is a significant relationship between a higher level of eHL and fewer reported barriers to mental health services (21). Nevertheless, the high-quality research about eHL's impact on attitude toward mental health help-seeking and wellbeing is insufficient in China (22), where there are nearly 900 million active Internet users.

**Figure 1:**
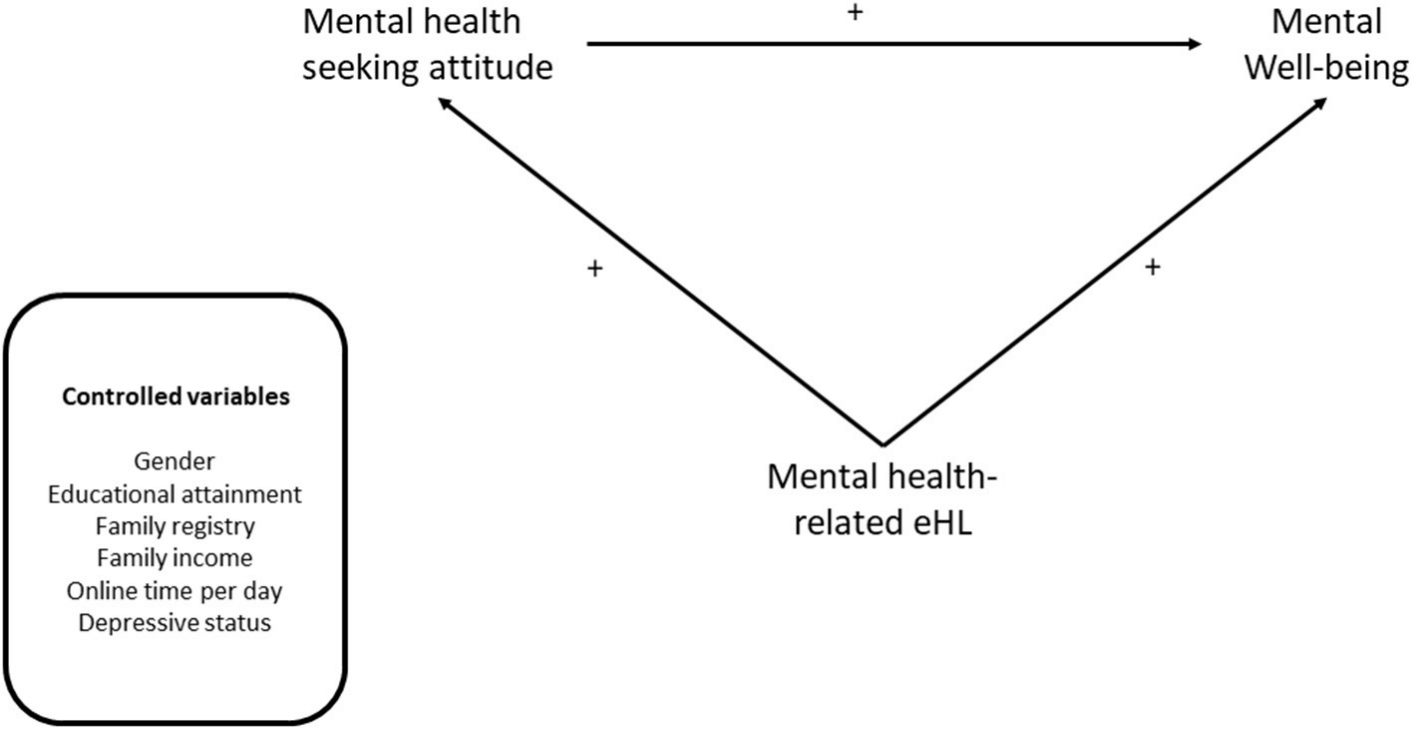
The conceptual model of this study.

Specifically, COVID-19 is identified as a catalyst for the development of China's online hospital ecosystem, medical industries, provision of health services, enhancement of overall service quality and efficiency by providing services through professional websites and social media (23). The eHL is important for empowering people to act upon their knowledge and enable them to take charge of their wellbeing. However, it is unclear to what extent the eHL is linked to mental health-seeking attitude and wellbeing in young electronic media users. Thus, this study aimed to understand the mechanism between them. Three major hypotheses are derived from the conceptual framework (Figure 1). According to Hypothesis 1, young electronic media users with a high level of eHealth literacy are more likely to show a positive attitude toward mental health help-seeking. Hypothesis 2 states that those with a high level of eHealth literacy are more likely to show enhanced mental wellbeing. Hypothesis 3 states That those with a positive attitude toward mental health help-seeking are more likely to show enhanced mental wellbeing.

## Methods

### Data and participants

A web-based cross-sectional survey investigating mental health status during COVID-19 was conducted using a non-probability snowball sampling method in October 2021 in Guangzhou, China. An online semi-structured questionnaire was developed using the “Wenjuanxin” platform, a professional surveying company, with an informed consent appended to it. The survey was initiated at a local university and the officers in charge of student affairs were approached to discuss the dissemination plan of the questionnaire. After confirmation, a link was sent to all students through the university's internal network using WeChat (a multipurpose messaging app). They were encouraged to share the link with their peers through their social network. All participants should be (a) 16~30 years old; (b) able to read Chinese; (c) able to provide informed consent. After they confirmed participation, a structured questionnaire with a sequential series of questions was presented. Respondents' information about their demographics, socioeconomic status, mental health status, and so on was collected. Given the targeted population was around 10 million [2021 Guangzhou Statistical Yearbook (http://tjj.gz.gov.cn/)], the margin of error was 3%, the confidence level was 95%, and the response distribution was 70%, a minimal sample size of 897 was determined. Finally, 1,008 valid responses were recorded. As the online surveying platform only saved the data about participants who completed the questionnaire, no response rate can be calculated. The study protocol and informed consent were approved by the Human Research Ethics Committee of the Hong Kong Polytechnic University (Ref No.: HSEARS20210328002). Written informed consent was obtained from all participants.

### Measures

#### Mental health related eHL

A modified eHealth literacy scale (eHEALS) was used to measure mental health-related eHL using the method introduced by Cormier et al. (20). The eHEALS, developed based on the concept of Web 1.0 (17), is one of the most widely used instrument to assess people's eHealth literacy worldwide. It comprises eight items rated on a five-point Likert scale with a sum score ranging between 8 and 40. A higher score indicates a greater perceived eHealth literacy. In this study, all items of the eHEALS were modified to specifically investigate people's eHealth literacy on mental health management (e.g., I know how to find helpful mental health resources on the Internet). Five university students were randomly selected to participate in the cognitive briefing to confirm the content validity of the modified instrument. The validity of the eHEALS in Chinese population has been assessed by Xu et al. (24). In this study, the Cronbach's alpha of the revised eHEALS was 0.96. The factor structure of the modified eHEALS is presented in the Appendix.

#### Mental help-seeking attitude

Mental Help-Seeking Attitudes Scale (MHSAS) was designed to measure respondents' overall evaluation of their seeking help from a mental health professional (25). It comprises nine items rated on a seven-point semantic differential scale. A higher score indicated a more positive attitude toward help-seeking. The English version of the MHSAS is provided by the developer and the research team has translated it into Chinese based on standard procedure (two forward and two backward translations, and five cognitive interviews). In this study, the Cronbach's alpha of the MHSAS was 0.88, the result of confirmatory factor analysis (1-factor structure) and item profile is provided in the Appendix.

#### Mental wellbeing

Short Warwick-Edinburgh Mental Wellbeing Scale (SWEMWS) is a short version of the Warwick-Edinburgh Mental Wellbeing Scale, developed to monitor mental wellbeing and evaluate the effectiveness of projects and policies to improve it. It uses seven statements of WEMWBS with a five-response category. A higher score indicates a better mental health status. The validity of the SWEMWS in Chinese population has been reported by Sun et al. (26). In this study, the Cronbach's alpha of the SWEMWS was 0.94.

#### Depression

Patient Health Questionaire-9 item (PHQ-9) is a multipurpose instrument for screening and measuring the severity of depression. It is the nine-item depression module from the complete PHQ. A score of five represents cut-off for respondents with or without depressive status. The validity of the PHQ-9 in Chinese population has been reported by Wang et al. (27). In this study, the Cronbach's alpha of the PHQ-9 was 0.9.

#### Control variables

The following control variables were involved in the analysis: gender (male = 0 and female = 1), educational attainment (secondary or below = 0, tertiary or above = 1), family registry (rural = 0, urban = 1), family income (below average = 0, equal to average = 1, above average = 2), daily Internet use time (≤3 h = 0, 4~6 h = 1; ≥7 h = 2), and depressive status (without = 0; with = 1).

### Statistical analysis

Descriptive analysis was used to describe the participants' background characteristics. Continuous and categorical variables were described using mean and standard deviation, and number and percentage, respectively. Structural equation modeling (SEM) was used to analyze the hypothesized relationship between eHL, mental health-seeking attitude, and wellbeing, and test the model fit. The robust weighted least square mean and variance adjusted estimator, which assumes non-normally distributed variables and provides the best option for modeling categorical or ordered data, was used (28). Three criteria were used to evaluate the goodness-of-fit of the model. They were (1) comparative fit index (CFI), values above 0.90 show a good model fit; (2) Tucker-Lewis index (TLI), values above 0.9 indicate a good model fit; and (3) the root mean square error of approximation (RMSEA), values <0.05 are equal to a “close fit” (29). R software was used to perform all data analysis. A *p*-value < 0.05 was identified as statistical significance.

## Results

Table 1 presents the respondents' characteristics. Around 29.8% were male, 94.8% were receiving tertiary level education, and 47.7% were rural residents. The mean age of the sample was 19.9 years [Standard deviation (SD) = 1.7], and more than half spend 4~6 h per day on information search and online social entertainment.

**Table 1:** Participants' characteristics (*n* = 1,008).

	***n***	**%**
**Sex**		
Male	300	29.8
Female	708	71.2
**Education**		
Secondary or below	53	5.2
Tertiary or above	955	94.8
**Family registry**		
Rural	481	47.7
Urban	527	52.3
**Perceived household income**		
Below local average	258	25.6
Equal to local average	671	66.6
Above local average	79	7.8
**Average time spent on internet per day**		
≤3 h	179	17.7
4~6 h	532	52.8
≥7 h	297	29.3
	**Mean (SD)**	**Range**
Age	19.9 (1.7)	16~27
PHQ-9 (depressive status)	4 (4.1)	0~27

Of the eight items of eHEALS, E6 “I have the skills I need to evaluate the mental health resources I find on the Internet” had the highest mean score (Mean = 3.51, SD = 0.93), while the lowest was seen in E8 “I feel confident using information from the Internet to make mental health decisions” (Mean = 3.34, SD = 0.94). The mean score for eHELAS was 27.3, which indicated a moderate level of eHL (Table 2).

**Table 2:** Participants' eHL, attitude for seeking mental health services, and mental wellbeing.

**Item**	**Mean/n**	**SD/%**	**Range**
**eHealth literacy**			
E1: I know how to find helpful mental health resources on the Internet	3.39	0.95	1–4
E2: I know how to use the Internet to answer my mental health questions	3.38	0.97	1–4
E3: I know what mental health resources are available on the Internet	3.41	0.97	1–4
E4: I know where to find helpful mental health resources on the Internet	3.48	0.95	1–4
E5: I know how to use the mental health information I find on the Internet to help me	3.44	0.94	1–4
E6: I have the skills I need to evaluate the mental health resources I find on the Internet	3.51	0.93	1–4
E7: I can tell high quality from low quality mental health resources on the Internet	3.38	0.96	1–4
E8: I feel confident in using information from the Internet to make mental health decisions	3.34	0.94	1–4
**Mental health seeking attitude**			
M1: seeking help from a mental health professional would be useful	5.94	1.29	1–7
M2: seeking help from a mental health professional would be important	4.95	2.17	1–7
M3: seeking help from a mental health professional would be useful	5.21	1.46	1–7
M4: seeking help from a mental health professional would be effective	5.61	1.37	1–7
M5: seeking help from a mental health professional would be good	5.31	1.73	1–7
M6: seeking help from a mental health professional would be healing	5.4	1.54	1–7
M7: seeking help from a mental health professional would be empowering	5.29	1.38	1–7
M8: seeking help from a mental health professional would be satisfying	5.16	1.52	1–7
M9: seeking help from a mental health professional would be desirable	5.26	1.46	1–7
**Mental wellbeing**			
W1: I've been feeling optimistic about the future	3.72	0.94	1–5
W2: I've been feeling useful	3.68	0.94	1–5
W3: I've been feeling relaxed	3.38	0.97	1–5
W4: I've been dealing with problems well	3.5	0.87	1–5
W5: I've been thinking clearly	3.67	0.85	1–5
W6: I've been feeling close to other people	3.55	1	1–5
W7: I've been able to make up my own mind about things	3.67	0.9	1–5
**Depressive status**			
No (<5)	632	62.7	
Yes (≥5)	376	37.3	

The overall attitude toward seeking mental health services was moderately positive (Mean = 5.34). Regarding MHSAS, respondents had the highest score for M1 “seeking help from a mental health professional would be useful” (Mean = 5.94, SD = 1.29) and the lowest for M2” seeking help from a mental health professional would be important” (Mean = 4.95, SD = 2.17). The overall mental wellbeing was moderate (Mean = 25.17). Regarding SWEMWS, respondents had the highest score for W1 “I've been feeling optimistic about the future” (Mean = 3.72, SD = 0.94) and the lowest score for W3 “I've been feeling relaxed” (Mean = 3.38, SD = 0.94). Over 37% indicated living with depressive status to some extent (Table 2).

The factor loadings of all variables were statistically significant. They ranged from 0.823 to 0.9 for eHEALs, 0.617 to 0.78 for MHSAS, and 0.794 to 0.86 for SWEMWS. The model fitted the data well with CFI = 0.975, TLI = 0.973, and RMSEA = 0.044, showing that mental wellbeing was measured well by the observed indicator variables (Table 3).

**Table 3:** Factor loadings of three measures.

**Item**	**Factor loading**	**95% C.I**.
E1	0.856^***^	0.828, 0.883
E2	0.874^***^	0.849, 0.898
E3	0.891^***^	0.866, 0.915
E4	0.88^***^	0.855, 0.906
E5	0.9^***^	0.876, 0.925
E6	0.851^***^	0.82, 0.883
E7	0.823^***^	0.79, 0.857
E8	0.849^***^	0.816, 0.882
M1	0.617^***^	0.557, 0.677
M2	0.463^***^	0.397, 0.528
M3	0.699^***^	0.651, 0.748
M4	0.68^***^	0.626, 0.734
M5	0.674^***^	0.621, 0.726
M6	0.724^***^	0.682, 0.766
M7	0.667^***^	0.605, 0.73
M8	0.777^***^	0.732, 0.822
M9	0.78^***^	0.74, 0.82
W1	0.832^***^	0.799, 0.866
W2	0.856^***^	0.819, 0.893
W3	0.794^***^	0.76, 0.827
W4	0.86^***^	0.834, 0.886
W5	0.874^***^	0.847, 0.9
W6	0.833^***^	0.804, 0.861
W7	0.843^***^	0.814, 0.873

*** p < 0.001.

**Figure 2:**
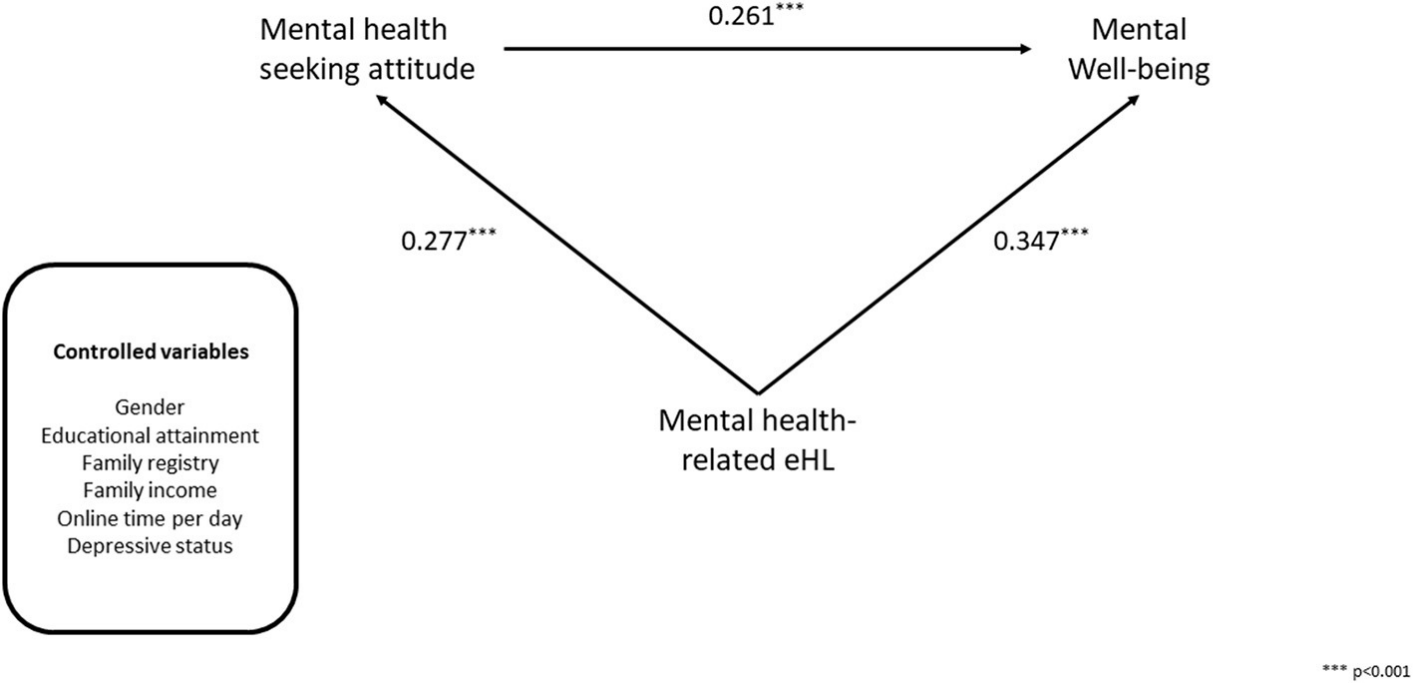
The standardized solution for significant paths of the structural model.

The unstandardized and standardized path coefficients for the structural model are demonstrated in Table 4. The standardized solution for significant paths of the structural model is presented in Figure 2. As hypothesized, the eHL showed a significant and direct effect on mental wellbeing. The higher the level of eHL, the better wellbeing of the participants (β = 0.347, *p* < 0.001). Mental health-seeking attitude is also positively correlated with mental wellbeing (β = 0.261, *p* < 0.001), indicating that the more positive attitude toward seeking mental health services, the better the participant's wellbeing. Moreover, the higher level of eHL can lead to a more significantly positive attitude toward seeking mental health service (β = 0.277, *p* < 0.001).

**Table 4:** Unstandardized and standardized path coefficients for SEM.

			**B**	**β**	**SE**	***p*** **-value**
Mental health seeking attitude	←	eHealth literacy	0.271	0.277	0.035	<0.001
Mental wellbeing	←	Mental health seeking attitude	0.255	0.261	0.034	<0.001
Mental wellbeing	←	eHealth literacy	0.332	0.347	0.036	<0.001
Mental wellbeing	←	Gender	0.055	0.032	0.031	0.29
Mental wellbeing	←	Educational attainment	0.239	0.068	0.027	0.01
Mental wellbeing	←	Family registry	0.072	0.046	0.03	0.13
Mental wellbeing	←	Online time per day	−0.068	−0.066	0.031	0.03
Mental wellbeing	←	Family income	0.089	0.063	0.031	0.04
Mental wellbeing	←	Depressive status	−0.085	−0.455	0.03	<0.001

Among the control variables, there was a positive relationship between educational level and mental wellbeing (β = 0.068, *p* = 0.01), which meant participants tend to report good mental wellbeing with a high educational level. Participants who spend a longer time on Internet have worse mental wellbeing (β = −0.066, *p* = 0.03). Decreased risk of developing depressive disorders predicts an increased probability of good mental wellbeing (β = −0.085, *p* < 0.001). However, gender and family registry exhibited no significant effects on participant's mental wellbeing (Table 4).

## Discussion

This study examined the association among mental health-related eHL, attitude toward seeking mental health services, and mental wellbeing in a sample of young electronic media users during COVID-19 in China. We found that those with a high level of eHL to search and use online mental health-related information tend to demonstrate a more positive attitude toward seeking mental health services and report better mental wellbeing. The Internet and its associated technologies are changing the nature of mental health practice (30), providing information through the Internet is identified as a cost-effective solution to improve mental health. This study provides empirical evidence that investments in digital health to improve young people's mental health related eHL could improve their mental wellbeing during and may even after the pandemic.

This study found a direct effect of increased eHL on improved mental wellbeing. Empirical evidence about a positive relationship between health or eHealth literacy and mental wellbeing is reported. For example, a Belgian study indicated that low HL levels act as a barrier to the integration of available online health resources and affect people's health outcomes (31). Xu et al. (32) found that patients with a high level of eHealth literacy are more likely to experience optimal decision-making processes and improved wellbeing among Chinese patients. Another study demonstrated that improving eHealth literacy may contribute to maintaining good psychological wellbeing of medical residents (33). However, few studies are investigating the impact of eHL on participants' mental wellbeing. Specifically, COVID-19 has negatively affected mental health and created new barriers for people with mental illnesses. Although online mental health services are highly encouraged and most participants use the Internet “almost constantly,” our study found that they scored lowest on the item regarding confidence to make mental health-related decisions. This is inconsistent with previous findings that online support and peer resources can increase patients' confidence and empowerment around decision-making (34). This may indicate that if we cannot ensure service users are equipped with sufficient eHL, the interventions' effectiveness to improve mental health might be compromised.

The mental help-seeking attitude was positively associated with eHL in this study. This shows that young people with higher knowledge and skills to analyze and use online mental health information are more likely to seek help from mental health professionals. The impact of eHL on help-seeking behavior is rarely reported, however, previous studies demonstrated inconsistent findings regarding web-based intervention's impact on the willingness to seek mental health help. For example, Taylor-Rodgers and Batterham (35) reported positive outcomes for help-seeking behaviors after web-based interventions are observed. Wright et al. (36) indicated that social media-based mental HL intervention is positively associated with mental health help-seeking behavior. Conversely, some studies reported no significant effect of web-based interventions on help-seeking attitudes, intentions, or behaviors (37–39). As several studies have confirmed that young adults are reluctant to seek professional help for common mental disorders because of several concerns, including cost, transportation, and confidentiality (40), our findings indicated that improving eHL could be a steppingstone to encourage health-seeking behaviors and improve people's mental health.

Although Internet use is particularly high for adolescents and young adults, the eHL level is not as high as expected. This extends the findings reported by a previous systematic review that young people's eHL skills are generally below average (41). This might be because most eHL-related interventions focus only on those who are using health care services, rather than those who need to access them. It is important to build education competencies among young electronic media users to improve their eHL, including mental health related eHL (42). Moreover, our findings demonstrated an adverse relationship between mental wellbeing and daily Internet use time, and that the association between daily Internet use time and eHL was statistically insignificant. Although empirical evidence has confirmed that Internet use is an important study topic for children and adolescents, its effects on wellbeing and mental health are ambiguous (43). This is partially consistent with previous findings that the quantity of screen time often does not equate with quality time spent with friends, family, colleagues, and communities (44). Previous studies also indicate that spending a long time online may “displace” time spent on social or cognitively stimulating activities, which could result in an increased risk of developing social isolation or depression (45). The eHL is a skill that should follow strict guidelines or training by professionals as online self-training courses might not be cost-effective.

Although eHEALS is a valid instrument to assess general eHL, problems in sensitivity might exist. Moreover, the eHEALS was mainly designed to assess the ability to seek and use online information to improve their healthcare (Web 1.0). Individuals' ability to engage in web-based intervention (Web 2.0) and even create online collective collaboration (Web 3.0) to improve mental health cannot be examined, which is the main use of the Internet in managing mental health. Empirical evidence has shown that anxiety, depression, and other psychological distress are highly prevalent among the general population during a pandemic (46). Thus, in the future, a mental health related eHL-specific instrument, which measure not only knowledge of Internet or computing, but also mental health literacy, such as recognition of disorders, knowledge of risk factors and causes, and knowledge of self-treatment, is needed to support the evaluation of individuals' ability to join in web-based mental health-related interventions to meet the challenges of a public health crisis.

Several limitations should be addressed. First, there might be a representative problem regarding our sample. In this study, all participants were recruited from a web-based survey, it might lead to selection bias that young people who are rarely used electronic media were not sufficiently included in this study. Additionally, more than 70% of participants were female, which may also compromise the representativeness of our sample. A heterogeneous sample should be collected in future studies. Second, the causal association between eHL, help-seeking attitude, and wellbeing cannot be established because of our cross-sectional design. The findings should be further examined by using longitudinal data. Third, as most participants reported normal mental health status, their request for mental health services or willingness of seeking knowledge might be low, which may affect the findings' validity. A group of young people with mental health problems should be invited to further assess whether a discord of findings exists. Last, mental health related eHL was assessed by a modified eHEALS. It has led to some critical components of mental health literacy cannot be sufficiently measured, which may affect the validity of our findings.

## Conclusion

This is the first study to assess the eHL level in a group of Chinese young electronic media users and confirm that it is significantly associated with their mental health-seeking attitude and wellbeing. We found that participants showed a moderate eHL level to manage their mental health during the pandemic and those with a high level showed a positive attitude toward seeking mental health support and reported a high level of mental wellbeing. We suggest that providing training to improve eHL may optimize young electronic media users' mental health outcomes. Moreover, the development of a mental health specific eHL instrument should be encouraged to measure a broad range of web-based mental health related knowledge, belief, self-esteem, problem-solving and social skills.

## Data availability statement

The raw data supporting the conclusions of this article will be made available by the authors, without undue reservation.

## Ethics statement

The studies involving human participants were reviewed and approved by Human Research Ethics Committee of the Hong Kong Polytechnic University. The patients/participants provided their written informed consent to participate in this study.

## Author contributions

RX: study concept and design, data analysis and interpretation, software, writing-original draft, and writing-review and editing. X-lB: data collection, software, and writing–review and editing. L-s-bS: data collection, visualization, and writing–review and editing. DW: study concept and design, provision of study materials or patients, collection and assembly of data, supervision, and writing–review and editing. All authors contributed to the article and approved the submitted version.
