# Supplementary material for: Associations between eHealth literacy, mental health-seeking attitude, and mental wellbeing among young electronic media users in China during the COVID-19 pandemic
Source: Front Public Health. 2023 Feb 24;11:1139786. doi: 10.3389/fpubh.2023.1139786 (PMC9998951; doi:10.3389/fpubh.2023.1139786)
Supplement: Supplementary file 1 [file Table_1.pdf]

Table A1 Results of CFA for Mental Help-Seeking Attitudes Scale

	CFI	TLI	RMSEA
One-factor model	0.979	0.969	0.079
	Factor loadings		
Item 1	0.55		
Item 2	0.52		
Item 3	0.62		
Item 4	0.62		
Item 5	0.71		
Item 6	0.76		
Item 7	0.61		
Item 8	0.81		
Item 9	0.82		

Table A2 Item profile for Mental Help-Seeking Attitudes Scale

	Item response (%)							Mean	SD	Item-total correlation
	1	2	3	4	5	6	7			
Item 1	1	2	2	9	10	34	42	5.9	1.3	0.59
Item 2	7	17	5	9	7	16	39	4.9	2.2	0.5
Item 3	1	2	9	25	14	25	24	5.2	1.5	0.69
Item 4	1	2	3	16	12	35	30	5.6	1.4	0.7
Item 5	2	10	7	11	9	30	31	5.3	1.7	0.76
Item 6	0	8	6	12	14	34	27	5.4	1.5	0.8
Item 7	1	3	4	24	17	30	22	5.3	1.4	0.61
Item 8	1	6	7	20	17	27	22	5.2	1.5	0.79
Item 9	1	4	7	20	15	30	23	5.3	1.5	0.79

Table A3 Result of CFA for MeHL

	CFI	TLI	RMSEA
One-factor model	0.98	0.97	0.031
	Factor loadings		
Item 1	0.86		
Item 2	0.88		
Item 3	0.9		
Item 4	0.89		
Item 5	0.89		
Item 6	0.83		
Item 7	0.81		
Item 8	0.85		
